# Impaired immunomodulatory ability of type 2 diabetic adipose-derived mesenchymal stem cells in regulation of inflammatory condition in mixed leukocyte reaction

**DOI:** 10.17179/excli2019-1575

**Published:** 2019-09-23

**Authors:** Sara Aliakbari, Mobin Mohammadi, Mohammad Ali Rezaee, Abbas Ali Amini, Shohreh Fakhari, Mohammad Reza Rahmani

**Affiliations:** 1 Student Research Committee, Kurdistan University of Medical Sciences, Sanandaj, Iran; 2 Department of Immunology and Hematology, Faculty of Medicine, Kurdistan University of Medical Sciences, Sanandaj, Iran; 3 Cancer and Immunology Research Center, Research Institute for Health Development, Kurdistan University of Medical Sciences, Sanandaj, Iran; 4 Zoonosis Research Center, Research Institute for Health Development, Kurdistan University of Medical Sciences, Sanandaj, Iran; 5 Department of Medical Laboratory Sciences, Faculty of Paramedical, Kurdistan University of Medical Sciences, Sanandaj, Iran

**Keywords:** type 2 diabetes, immunomodulation, mesenchymal stem cells, adipose tissue, inflammation

## Abstract

The immunomodulatory properties of type 2 diabetic patients' adipose-derived mesenchymal stem cells (D-ASCs) has not been extensively studied. In this study, we compared the immunomodulatory properties of D-ASCs and non-diabetic subjects mesenchymal stem cells (ND-ASCs) in co-culture with mixed leukocyte reaction (MLR). ASCs were isolated from adipose tissue samples of type 2 diabetic and non-diabetic subjects (age: 40-55). D-ASCs and ND-ASCs were co-cultured with two-way MLR. Peripheral blood mononuclear cells (PBMCs) proliferation ratio, protein levels of IFN-γ and IL-10, mRNA expression of *COX-2*, *TNF-α*, *TGF-β1* and *IL-6* genes in MLR, D-ASCs and ND-ASCs co-cultures were assessed using XTT, ELISA and Real-time qRT-PCR, respectively. PBMCs proliferation on days 2 and 4 of D-ASCs co-culture was higher than ND-ASCs co-culture of the same days (*p* < 0.001). IFN-γ level decreased on day 4 compared to day 2 of ND-ASCs co-culture, but its level had not changed in D-ASCs co-culture. *COX-2* expression on days 2 and 4 of D-ASCs co-culture was lower than ND-ASCs co-culture of the same days (*p* < 0.05). The expression of *TNF-α* and *IL-6 *on days 2 and 4 of D-ASCs co-culture were higher than ND-ASCs co-culture of the same days (*p* < 0.001). *TGF-β1* on day 4 of ND-ASCs co-culture showed a slightly higher expression than D-ASCs co-culture of the same day. Lower suppression of PBMCs proliferation, declined expression of anti-inflammatory and upregulated expression of pro-inflammatory factors in D-ASCs co-culture, indicated an impairment of these cells in modulation of the inflammatory condition.

## Introduction

Diabetes mellitus is one of the most common chronic metabolic diseases (American Diabetes Association, 2009[3]). In 2015, more than 400 million people were living with diabetes worldwide, which is estimated to increase to 592 million by 2035 (Guariguata et al., 2014[17]). Diabetes is characterized by increased plasma glucose levels, due to degeneration of Langerhans islets in type 1 and insulin resistance in type 2 (American Diabetes Association, 2009[3]). Type 2 diabetes is a chronic systemic inflammatory disease in which inflammatory responses play a role in defective glucose uptake by adipose tissue, liver and skeletal muscle, leading to impaired regulation of gluconeogenesis and glycogenolysis (Kohlgruber and Lynch, 2015[28]).

Despite the structural and histological simplicity, adipose tissue is dynamic and pleiotropic, which is a rich source of mesenchymal stem cells (MSCs). However, it has immunologic and endocrine activity, capable of receiving and sending signals to regulate energy reserves, appetite, inflammation and immune system (Hatting et al., 2018[19]). Therefore, adipose tissue expansion is an important risk factor for all chronic obesity-related diseases, including type 2 diabetes. Following functional impairment of adipose tissue, ectopic visceral fat generates, which is identified by cellular changes, increased fat reserves and decreased insulin sensitivity in adipocytes, and secretion of pro-inflammatory cytokines and diabetes-related adipokines (Bluher, 2009[4]). Reports indicated that in excessive obese individuals, differentiation of adipose-derived MSCs (ASCs) into adipocytes significantly increases and therefore plays an important role in the maintenance of adipose tissue mass and function (Onate et al., 2013[35]; Maumus et al., 2011[33]). 

Immunomodulatory properties are one of the main characteristics of ASCs, and they have gained extensive interest due to their relative ease of isolation and potential for allogeneic and autologous transplantations in inflammatory diseases (Frese et al., 2016[16]). On the other hand, understanding that the immune system can be effective in regulation of metabolic pathways (Pearce and Pearce, 2013[38]) presents a new way of thinking about diabetes and its management. Different characteristics of diabetic ASCs have been studied. Impairment of ASCs angiogenic, differentiation and survival potentials have been reported in diabetic patients (Ferrer-Lorente et al., 2014[15]). Culture of human ASCs in high glucose medium caused an increment in reactive-oxygen species, and therefore impaired pro-angiogenic and proliferative capacity of these cells (Kim et al., 2008[25]). In an excisional wound splinting model of diabetic mice, the expression of interleukin-6 (*IL-6*) and tumor necrosis factor alpha (*TNF-α*) upregulated in the diabetic MSCs. The data revealed stem cell impairment as a major complication of type 2 diabetes in mice and suggested that the disease may stably alter endogenous MSCs (Shin and Peterson, 2012[43]). In the mouse model of diabetic wound receiving diabetic human ASCs, high expression of early growth response protein 1 (*EGR-1*) as an insulin signaling mediator, along with inflammation, led to increased production of IL-6 and transforming growth factor-beta 1 (TGF-β1). The result showed a reduction in the therapeutic potential of diabetic ASCs compared to non-diabetics ASCs (Trinh et al., 2016[46]). However, thus far, the immunomodulatory properties of type 2 diabetic patients ASCs in inflammatory models has not been compared to non-diabetic subjects. The aim of this study was to compare the immunomodulatory properties of ASCs isolated from diabetics (D-ASCs) to non-diabetic individuals (ND-ASCs) in mixed leukocyte reaction (MLR).

## Materials and Methods

### Isolation and expansion of ASCs

Samples of omental adipose depots were obtained from non-diabetic (age: 40-55 years old, fasting plasma glucose (FPG) < 100 mg/dl) and type 2 diabetic subjects (age: 40-55 years old, FPG ≥ 126 mg/dl), who had undergone laparoscopic surgery, after their signed and informed written consent. Non-diabetic samples were obtained from subjects without any background of diabetes, neither other metabolic diseases, which had normal FPG (FPG < 100 mg/dl). We isolated MSCs from adipose tissues of 7 diabetic and 7 non-diabetic subjects by a non-enzymatic method. Briefly, Hanks' balanced salts solution (HBSS) containing penicillin (300 U/ml), streptomycin (300 μg/ml) and amphotericin B (25 μg/ml) was used to transfer and wash the samples. To isolate ASCs, the samples were cut into smaller pieces (≈5 mg) and placed in 6-well cell culture plates (SPL, Korea). Subsequently, the surface of each adipose tissue piece was covered with 50 μl fetal bovine serum (FBS) (Life Technologies, UK). The culture plates were incubated for 24 hours at 37 °C in a humidified atmosphere containing 5 % CO_2_. After this, FBS was removed and low glucose Dulbecco modified Eagles media (DMEM-LG) (Life Technologies, UK) containing streptomycin (100 μg/ml), penicillin (100 U/ml) and 15 % FBS (complete culture media) were added to the wells. The wells were monitored daily by an inverted microscope until the spindle-shaped fibroblast cells appeared in the margin of adipose tissue pieces. The culture media were replaced 3 times a week until the cells reached 70-80 % confluency. Then they were harvested with trypsin/EDTA solution and expanded for 4 sequential passages.

### Flow cytometry

Isolated cells from adipose tissue samples of each diabetic and non-diabetic subjects were trypsinized at passage 4, pelleted and suspended in FCM buffer (phosphate-buffered saline (PBS) containing 0.5 % Bovine Serum Albumin). The immunophenotyping of D-ASCs and ND-ASCs was performed using conjugated antibodies, including CD105-PE, CD34-PE, CD45-FITC, CD73-FITC, and CD90-PerCP (eBioscience, USA). Appropriate isotype-matched control mouse antibodies for PE, FITC and PerCP (eBioscience, USA) were used in all analyses. The cells were incubated with 10 µl of each antibody or isotype antibody for 45 min at 4 °C. After incubation time, cells were washed three times with FCM buffer, fixed with 1 % paraformaldehyde and subjected to flow cytometry (FACS Calibur, Becton Dickinson, USA). Data of flow cytometry were analyzed by the FLOWJO V.7.6 software (FLOWJO, LLC, USA). 

### Adipogenic differentiation

3×10^4^ cells/cm^2^ of each diabetic and non-diabetic adipose tissue-derived cells were seeded in a 24-well plate (SPL, Korea). After reaching 80 % confluency, the culture media were replaced with the adipogenic differentiation media (Life Technologies, USA). The differentiation media were changed every 3 days. After 21 days, the cells were washed with PBS and fixed with 10 % formalin. Then, cells were stained with Oil Red O solution and checked by an optical microscope (Olympus, Japan) to observe the fat vacuoles.

### Osteogenic differentiation

5×10^3^ cells/cm^2^ of each diabetic and non-diabetic adipose tissue-derived cells were cultured in a 24-well plate with complete culture media. After 24 hours, the culture media were replaced with osteogenic differentiation media (Life Technologies, USA). The differentiation media were changed every 3 days. After 21 days, the cells were washed with PBS and fixed with 10 % formalin. To determine calcium deposits, cells were stained with 2 % Alizarin Red S solution and visualized by an optical microscope.

### Chondrogenic differentiation

At first, a cell suspension containing 1.6×10^7^ cells/ml of each diabetic and non-diabetic adipose derived cells were prepared. Then 5 μl droplets of cell suspension were placed in the center of a 96-well microplate and incubated for 2 hours at 37 °C. After that, chondrogenic differentiation media (Life Technologies, USA) were added to the wells. The media were replaced every 3 days. After 14 days, wells were washed with PBS and fixed with 10 % formalin. To investigate proteoglycan production, cells were stained with Alcian Blue 1 % in 0.1 N HCl and visualized by an optical microscope.

### Co-culture and leukocyte proliferation assay

The effect of non-diabetic and diabetic ASCs on peripheral blood mononuclear cells (PBMCs) proliferation was investigated in the MLR model. For ASCs/MLR co-culture, initially, 5×10^3^ of each non-diabetic and diabetic ASCs were seeded in separate wells of a 96-well microplate. After 24 hours, the proliferation of these cells was inhibited by 10 μg/ml mitomycin C. 5 ml of anticoagulant-treated blood specimens were collected from two healthy donors separately, and PBMCs were isolated with Ficoll-Paque PREMIUM (1.077g/ml) (GE Healthcare, Life Sciences, UK) density gradient centrifugation. To perform co-culture, 10^5^ PBMCs were isolated from each donor and were added to the co-culture wells containing D-ASCs or ND-ASCs. Also, 10^5^ PBMCs of each donor were added to the MLR wells. The plate was incubated at 37 °C for 4 days. The PBMCs proliferation activity was measured on days 0, 2, and 4 of D-ASCs/MLR, ND-ASCs/MLR co-cultures, and MLR groups, using XTT colorimetric method according to the manufacturer's instruction (PromoCell GmbH, Germany). The proliferation ratio was calculated on days 0, 2 and 4 of the co-culture groups toward MLR of the same days using the following equation:

Proliferation ratio: (Co-culture-OD/MLR-OD) × 100

Then, the proliferation ratio on day 0 of D-ASCs/MLR and ND-ASCs/MLR co-cultures were considered 100 %. The normalized proliferation ratio on days 2 and 4 of the co-culture groups was calculated as follows:

Normalized proliferation ratio: Proliferation ratio of _day 2 or 4 _× 100/proliferation ratio _day 0_

### RNA extraction and real-time quantitative reverse transcription PCR (RT-qPCR)

To evaluate the expression level of cyclooxygenase-2 (*COX-2*), *TNF-α*, *TGF-β1* and *IL-6* genes in MLR, D-ASC/MLR and ND-ASC/MLR co-cultures, cells were cultured in 24-well plates. For the co-culture, initially 5×10^4^ D-ASCs or ND-ASCs were seeded in separate wells. After 24 hours, the proliferation of these cells was inhibited by 10 μg/ml mitomycin C. PBMCs were isolated from blood samples of two healthy donors using Ficoll-paque PREMIUM. To perform co-culture, 6×10^5 ^PBMCs from each donor were added to the D-ASCs/MLR and ND-ASCs/ MLR co-culture wells. Also, for MLR, the same numbers of PBMCs from each donor were added to the MLR group wells. Plates were incubated at 37 °C for 4 days. The total RNA was extracted from PBMCs on days 2 and 4 of all groups using the phenol-chloroform method according to the manufacturer's protocol (Pars-Tous Biotechnology, Iran). 1 μg of the RNA was used to synthesize cDNA, according to the kit instructions (Pars-Tous Biotechnology, Iran). The mRNA levels of *COX-2*, *IL-6*, *TNF-α* and *TGF-β1* genes were evaluated using specific designed primers (Table 1(Tab. 1)) and SYBR Premix EX taq II (Takara, Japan) in the Rotor-Gene TM 6000 sequencer detection system (Corbett Life Science, Australia). *β-actin* gene was used as internal control and target genes expression in the MLR group were used as external control. The relative expression of target genes in ND-ASCs/MLR and D-ASCs/MLR groups were calculated using the 2^-ΔΔCT ^formula.

ΔΔCT = ΔCT _co-culture _(CT_ target gene - _CT *_β-actin_*) - ΔCT _MLR_ (CT _target gene_ - CT *_β-actin_*)

### Enzyme linked-immunosorbent assay (ELISA)

In order to evaluate the levels of IL-10 and IFN-γ in D-ASCs/MLR, ND-ASCs/MLR co-cultures, and MLR groups, the supernatant of cultures on days 2 and 4 of experiments were separately collected from 24-well plates and stored at -70 °C. The level of these cytokines was quantified by ELISA kits according to the manufacturer's instruction (Mabtech, Inc., Sweden).

### Statistical analysis

Data were analyzed using SPSS V.16 software (IBM Analytics, USA). Data of proliferation, gene expression, and cytokines levels were analyzed in all study groups with Mann-Whitney U test and *p *< 0.05 was considered as the significance level.

## Results

### Characterization of ND-ASCs and D-ASCs

**Figure 1:**
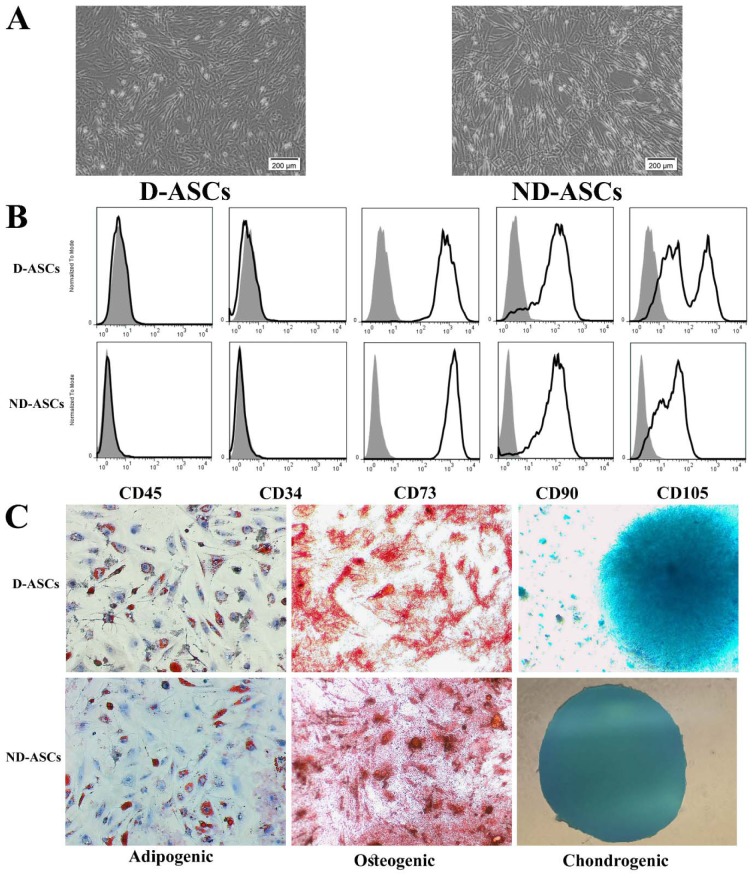
Characterization of ND-ASCs and D-ASCs. (A) Representative inverted microscope image of ND-ASCs and D-ASCs at passage 4 demonstrated the spindle-shaped morphology of both cells. 40x magnification, (B) Tri-lineages differentiation of ND-ASCs and D-ASCs. Isolated cells from passage 4 were respectively induced into osteogenic, adipogenic, and chondrogenic lineages in specific differentiation media of each lineage. 40x magnification, (C) ND-ASCs and D-ASCs at passage 4 were positive for the MSC-related surface markers of CD105 (PE), CD90 (PerCP) and CD73 (FITC), while they were both negative for CD45 (FITC) and CD34 (PE), in flow cytometry analysis. Abbreviation: D-ASCs: Diabetic adipose derived stem cells; ND-ASCs: Non-diabetic adipose derived stem cells; MSC: Mesenchymal stem cell

The isolated cells from diabetic and non-diabetic adipose tissue samples showed plastic adherent and spindle-shaped morphology (Figure 1A(Fig. 1)). In addition, both of these cells differentiated into osteogenic, adipogenic and chondrogenic lineages, which were confirmed by specific staining (Figure1B(Fig. 1)). Flow cytometry analysis of isolated cells at passage 4 showed that both isolated cells from diabetic and non-diabetic subjects' adipose tissue were positive for CD90, CD73, and CD105, and negative for CD45 and CD34 markers (Figure 1C(Fig. 1)).

### Impaired ability of D-ASCs in suppression of PBMCs proliferation

**Figure 2:**
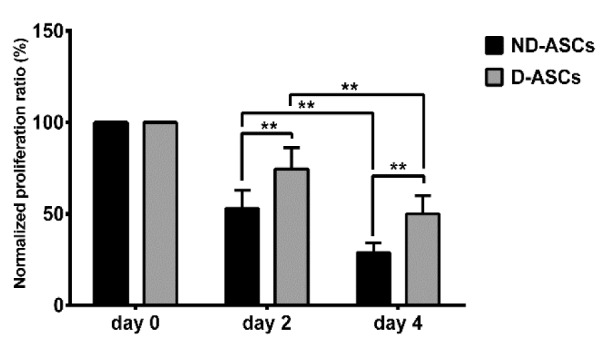
Effects of ND-ASCs and D-ASCs on PBMCs proliferation in the two-way mixed leukocyte reaction (MLR). Mitotically inactivated ND-ASCs, and D-ASCs were cultured with PBMCs from two donors, PBMCs proliferation was assessed on days 0, 2 and 4 of the experiment using XTT. Data are presented as mean ± SD normalized proliferation ratio of ND-ASCs and D-ASCs on days 2 and 4 of the experiment. Normalized proliferation ratio: Proliferation ratio of day 2 or 4 × 100/proliferation ratio day 0, (the proliferation ratio on day 0 of D-ASCs/MLR and ND-ASCs/MLR were considered as 100 %). ND-ASCs and D-ASCs suppressed proliferation of PBMCs (P < 0.001). The normalized proliferation ratio of PBMCs on days 2 and 4 of the DASCs co-culture were significantly higher than the ND-ASCs co-culture of the same days (P < 0.001). ** indicates a significance level of below 0.01. Abbreviation: D-ASCs: Diabetic adipose derived stem cells; NDASCs: Non-diabetic adipose derived stem cells; PBMC: Peripheral blood mononuclear cell

PBMCs proliferation was suppressed in the presence of ASCs on days 2 and 4 of D-ASCs/MLR and ND-ASCs/MLR co-cultures. The normalized proliferation ratio of PBMCs on day 2 of ND-ASCs and D-ASCs co-cultures were 52.88 ± 9.9 % and 74.53 ± 11.6 %, respectively, which reduced to 28.75 ± 5.4 % and 49.98 ± 9.84 % on day 4. The reduction of PBMCs proliferation between days 2 and 4 was significant in both co-culture groups (*p* < 0.001). The normalized proliferation ratio of PBMCs on days 2 and 4 of the D-ASCs co-culture were significantly higher than the ND-ASCs co-culture of the same days (*p* < 0.001) (Figure 2(Fig. 2)).

### D-ASC co-culture increased the TNF-α and IL-6 levels and decreased COX-2 level

**Figure 3:**
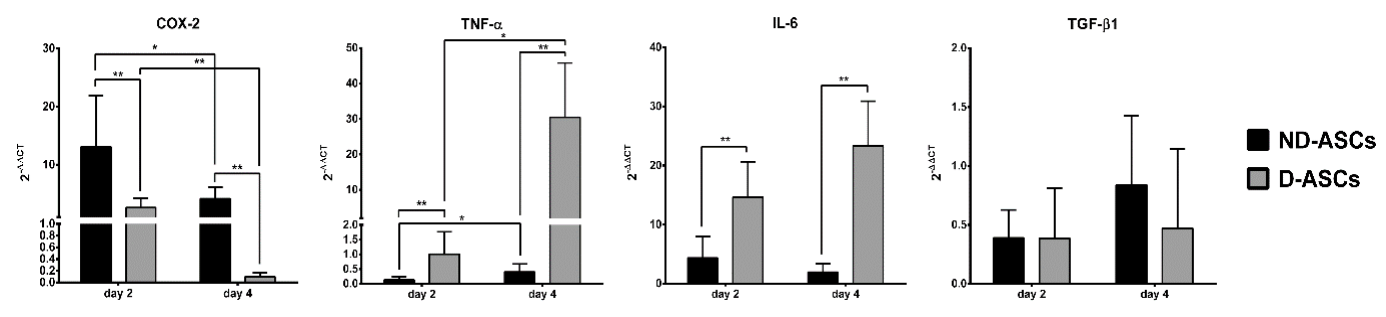
Relative expression of COX-2, TNF-α, IL-6 and TGF-β1 genes in ND-ASCs/PBMCs and D-ASCs/PBMCs co-cultures evaluated by Real-time RT-qPCR method. To perform Real-time RT-qPCR, target genes expression in the MLR group was considered as the exogenous reference, also, β-actin gene expression was considered as endogenous reference. The genes expression was calculated using 2 ^-∆∆CT^ formula: ΔΔCT = ΔCT co-culture (CT target gene - CT β-actin) - ΔCT MLR (CT target gene - CT β-actin). Data are presented as mean ± SD 2 ^-∆∆CT^ amounts. COX-2 expression on days 2 and 4 of D-ASCs co-culture was lower than ND-ASCs co-culture on the same days (P < 0.001). TNF-α and IL-6 expressions on days 2 and 4 of D-ASCs co-culture were higher than ND-ASCs co-culture on the same days (P < 0.001). There was no significant difference in the expression of TGF-β1 between both groups (P > 0.05). * Indicates a significance level below 0.05 and ** indicates a significance level of below 0.01. Abbreviation: D-ASCs; Diabetic adipose derived stem cells, ND-ASCs: Non-diabetic adipose derived stem cells; PBMC: Peripheral blood mononuclear cell; MLR: Mixed leukocyte reaction; COX-2: Cyclooxygenase-2; TNF-α: Tumor necrosis factor alpha; IL-6: interleukin-6; TGF-β1: transforming growth factor-beta

The mRNA levels of *COX-2*, *TNF-α*, *IL-6* and *TGF-β1* genes on days 2 and 4 of MLR, ND-ASCs/MLR and D-ASCs/MLR co-cultures were evaluated by real-time RT-qPCR. The relative mRNA expression of these genes between the studied groups was compared using 2^-ΔΔCT^ formula. *COX-2* expression on day 2 of ND-ASCs/MLR and D-ASCs/MLR was 13.1 ± 8.8 and 2.62 ± 1.6 respectively, which decreased to 4.15 ± 2.03 (p = 0.03) and 0.1 ± 0.06 (p = 0.001) on day 4. The expression level of *COX-2* on days 2 and 4 of D-ASCs co-culture was significantly lower on the same days of ND-ASCs co-culture (*p* < 0.05). *TNF-α* expression on day 2 of ND-ASCs co-culture was 0.134 ± 0.1, and in D-ASCs co-culture was 1.006 ± 0.75, which increased to 0.399 ± 0.27 (*p* = 0.03) and 30.47 ± 15.33 (*p* = 0.01) on day 4, respectively. This gene expression on days 2 and 4 of D-ASCs co-culture was higher than the same days of ND-ASCs co-culture (*p* < 0.01). The *IL-6* expression on day 2 of ND-ASCs co-culture was 4.33 ± 3.68 and in D-ASCs co-culture was 14.66 ± 5.98, which altered to 1.93 ± 1.47 (P = 0.24) and 23.33 ± 7.56 (*p* = 0.06) on day 4, respectively. This gene had a higher expression in D-ASCs co-culture compared to ND-ASCs co-culture on both days of experiment (*p* < 0.05). The expression of *TGF-β1* on day 2 of ND-ASCs and D-ASCs co-cultures was 0.389 ± 0.238 and 0.384 ± 0.427, respectively, and it changed to 0.839 ± 0.59 and 0.47 ± 0.673 on day 4. There was no significant difference in the expression of this gene in the two groups, nor in the two days of the experiment (Figure 3(Fig. 3)).

### Effects of ND-ASCs and D-ASCs on IL-10 and IFN-γ levels

**Figure 4:**
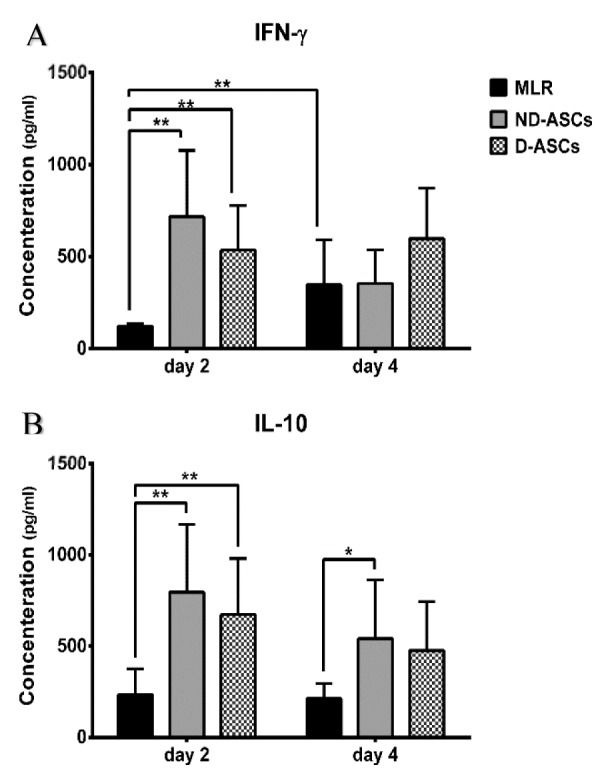
Effects of ND-ASCs and D-ASCs on IL-10 and IFN-γ levels. IL-10 and IFN-γ levels were assessed in supernatant of MLR and co-cultures on days 2 and 4 of experiment using ELISA. Mean of cytokines concentrations were compared in co-cultures and MLR groups. (A) IFN-γ levels increased in ND-ASCs and D-ASCs co-cultures compared to MLR on day 2. This cytokine level declined in ND-ASCs co-culture on day 4 but didn't change in D-ASCs co-culture, compared to day 2 of the same groups. (B) IL-10 levels were higher in the ND-ASCs and D-ASCs co-cultures compared to the MLR group on both days of experiment. Also, its level decreased on day 4 of co-culture groups compared to day 2 of the same groups. However, there was no statistically significant difference between D-ASCs and ND-ASCs co-culture on both days of the experiment (p > 0.05). * Indicates a significance level below 0.05 and ** indicates a significance level of below 0.01.

IFN-γ and IL-10 levels in supernatants of ND-ASCs/MLR, D-ASCs/MLR co-cultures and MLR were measured on days 2 and 4 of the experiment by ELISA. Generally, on day 2 of both co-culture groups, IFN-γ and IL-10 levels increased compared to the same days of MLR (*p* < 0.05). The IFN-γ level on day 2 of ND-ASCs and D-ASCs co-cultures were 717.66 ± 359.80 pg/ml and 536 ± 241.50 pg/ml, respectively, which reached 354.14 ± 182.35 pg/ml and 597.66 ± 274.30 pg/ml on day 4. The level of this cytokine reduced on day 4 compared to day 2 in the ND-ASCs co-culture, while there was a slight increase in this cytokine level in D-ASCs co-culture. However, these changes were not statistically significant. The IL-10 level on day 2 of ND-ASCs and D-ASCs co-cultures were 796 ± 371.14 pg/ml and 672.66 ± 308.05 pg/ml, respectively, which reduced to 541.14 ± 322.33 pg/ml and 476 ± 268.05 pg/ml on day 4. The level of this cytokine decreased on day 4 of both co-culture groups. The IL-10 level on days 2 and 4 of D-ASCs co-culture was slightly lower than ND-ASCs co-culture. However, these changes were not statistically significant (Figure 4(Fig. 4)).

## Discussion

Diabetes underlie factors such as diet, stress and genetic, can provide an inflammatory condition by changing the adipose tissue homeostasis, that in a vicious cycle, the exacerbation of this inflammation causes hyperglycemia and insulin resistance (Jeevendra Martyn et al., 2008[23]). Since ASCs play an important role in adipose tissue homeostasis, above-mentioned event can lead to their impaired function. Previous studies have reported the impairment of isolated ASCs from diabetic patients in different aspects (Cianfarani et al., 2013[8]). In the present study, we compared the immunomodulatory properties of isolated ASCs from diabetic and non-diabetic subjects. Other and our previous *in vitro* and *in vivo* studies showed that MSCs reduce the proliferation of immune cells in inflammatory condition (Abolhasani et al, 2018[1]; Kovach et al., 2015[29]). This feature is considered as one of the main indicators of MSCs immunomodulatory properties. The results of the present study showed that PBMCs proliferation was suppressed on days 2 and 4 of both ND-ASCs/MLR and D-ASCs/MLR co-cultures, but the normalized proliferation ratio of PBMCs in the ND-ASCs co-culture was significantly lower than D-ASCs co-culture in both days of experiment, which indicates an impairment in suppressing cell proliferation by D-ASCs.

MSCs have an adaptable function to their microenvironment. Presence of pro-inflammatory cytokines such as IFN-γ in MSCs microenvironment affects the immunomodulatory properties of these cells (Kadle et al., 2018[24]). At low levels of IFN-γ, MSCs acquire veto function; increase MHC-II expression on their surface and gain the function of antigen-presenting cells (APCs). In a different manner, the increment of IFN-γ decreases MHC-II expression on MSCs surface, which induces the immunosuppressive phenotype of these cells (Chan et al., 2006[5]). In the present study, IFN-γ level increased significantly on day 2 of both co-culture groups compared to MLR. Increased level of IFN-γ on day 2 could license ASCs to inhibit immune cells, which was aligned with decreased proliferation of PBMCs in both groups of co-cultures. This cytokine level on day 2 of ND-ASCs co-culture was higher than D-ASCs co-culture, which could lead to the better licensing of ND-ASCs for inhibitory function compared to D-ASCs. The proliferation results also indicated the better suppression of PBMCs proliferation in ND-ASCs co-culture, which was along with the higher level of INF-γ in the ND-ASCs co-culture. In previous studies, we showed that IFN-γ level decreases in the final days of MSCs/MLR co-culture (Abolhasani et al., 2018[1]; Hesami et al., 2017[21]).

Also, in the present study, this cytokine level decreased on day 4 of ND-ASCs/MLR co-culture. Contrary to these observations, the level of this cytokine did not decrease on day 4 of D-ASCs/MLR co-culture. *In vivo* studies have shown that in inflammatory conditions and autoimmune diseases, MSCs can decrease the level of pro-inflammatory cytokines, such as IFN-γ (DelaRosa et al., 2012[12]). The higher level of IFN-γ on day 4 of D-ASCs co-culture may suggest an impairment in the licensing of these cells to the immunosuppressive phenotype.

IL-10 is an inhibitory and important cytokine in MSCs immunomodulation. These cells secrete IL-10 in an inflammatory environment and in presence of IFN-γ, IL-1β, and TNF-α (Domenis et al,, 2018[13]). On the other hand, MSCs produce factors that induce secretion of IL-10 by PBMCs, macrophages and tolerogenic DCs (Ma et al., 2014[31]). In the present study, the higher level of IL-10 on day 2 of both co-cultures compared to MLR indicates the role of ASCs in induction of this cytokine secretion. Although, level of IL-10 was not significantly different in the D-ASCs and ND-ASCs co-cultures, but its level in D-ASCs co-culture was lower than ND-ASCs, which could attribute to impaired immunomodulatory ability of D-ASCs.

The cyclooxygenase enzyme has two isoforms that play an important role in biosynthesis of prostaglandins from arachidonic acid. COX-1 is constitutively expressed in many tissues, and COX-2 is an inducible enzyme that cannot be measured in normal conditions and resting immune cells. COX-2 is induced during inflammation and immune responses that results in production of prostaglandin E2 (PGE2) (Pablos et al.,1999[36]). Macrophages express *COX-2,* hence they are rich sources of prostaglandins (Thivierge and Rola-Pleszczysnki, 1995[44]). Production of this enzyme is effective in the polarization of macrophages to anti-inflammatory M2 phenotype (Na et al., 2013[34]). Prostaglandins, in particular, their E series, are important pleiotropic immunomodulatory molecules (Chou et al., 2014[7]). These molecules increase secretion of IL-10 by macrophages through prostaglandin E2 receptor 2 (EP2) and prostaglandin E2 receptor 4 (EP4) (MacKenzie et al., 2013[32]). As mentioned previously, MSCs are licensed with help of specific molecules to perform their immunomodulatory function. The inflammatory microenvironment (high levels of IFN-γ) can induce *COX-2* and *IDO*, which are prerequisite of MSCs licensing to immunosuppressive phenotype (Yagi et al., 2010[49]). Crop et al. reported that up-regulation of *COX-2* in the ASCs/MLR co-culture could indicate the role of PGE2 in enhancement of MSCs immunosuppressive capacity (Crop et al., 2010[11]). The importance of IFN-γ-induced IDO and PGE2 in suppression of lymphocytes proliferation has been studied (Ryan et al, 2007[41]). In IFN-γ-treated MSCs/B cells co-cultures, expression of *IDO* and *COX-2* from MSCs increased, and *COX-2* inhibition led to suppression of IL-10 secretion (Hermankova et al., 2016[20]). Aggarwal and Pittenger (2005[2]) showed that in human MSCs/T cells co-culture, MSCs increased PGE2 synthesis. Moreover, they showed that PGE2 inhibitors decreased the immunomodulatory properties of MSCs. In co-culture of bone marrow MSCs (BM-MSCs)/MLR, Hsu et al. (2013[22]) discovered the role of COX-2/PGE2 axis in the induction of cells with a phenotype similar to Tr_1_. These cells were CD4^+^IL-10^+^IFN-γ^+^ and suppressed MLR proliferation through IL-10 production. In the present study, *COX-2* expression on days 2 and 4 of D-ASCs co-culture was remarkably lower than ND-ASCs co-culture. The expression of this enzyme on day 2 of the ND-ASCs co-culture was at its highest level, which was along with the highest levels of IFN-γ and IL-10 on the same day, and it was in consistence with the results of Hus et al. (2013[22]). Simultaneous high levels of IL-10 and IFN-γ, besides significant expression of *COX-2*, can attribute to the higher immunomodulatory and proliferation suppression ability of ND-ASCs compared to D-ASCs.

Senescent cells have a specific secretory phenotype called 'senescence-associated secretory phenotype (SASP)', which is accompanied by production of pro-inflammatory cytokines, proteases and growth factors from these cells (Coppé et al., 2010[9]). Studies have shown that senescence in human MSCs causes secretion of inflammation exacerbating factors such as TGF-α and IFN-γ, and reduces the immunomodulatory activity of these cells (Turinetto et al., 2016[47]). Diabetic ASCs in presence of elevated glucose concentration and daily amounts of TNF-α showed a higher level of cellular senescence and apoptosis compared to non-diabetic ASCs (Cramer et al., 2010[10]). In this regard, it has been observed that exposure of ASCs to high glucose levels leads to the induction of senescence in these cells (Kim et al., 2012[26]). Senescence in MSCs is associated with reduced synthesis of COX-2 and PGE2, and decreased immunomodulatory ability of these cells (Yu et al., 2014[50]). In the present study, the expression of* COX-2* on both days of the experiment in D-ASCs co-culture was far lower than ND-ASCs co-culture. It is likely that lower expression of *COX-2* in D-ASCs co-culture results from the exposure of these cells to hyperglycemia and subsequent induction of senescence in diabetic ASCs.

IL-6 with a dual pro/anti-inflammatory function plays a key role in immune responses, inflammation, cell survival, apoptosis, and cell proliferation (Scheller et al., 2011[42]). This cytokine, along with *TGF-β1*, contributes to regulation of the balance between T cells differentiation toward pro-inflammatory Th17s or anti-inflammatory Tregs (Scheller et al., 2011[42]; Kimura and Kishimoto, 2010[27]). BM-MSCs limits the secretion of IL-6 by macrophages, and alters condition toward induction of anti-inflammatory macrophages (Vasandan et al., 2016[48]). Our results indicated higher expression of *IL-6* in D-ASCs co-culture compared to ND-ASCs co-culture. Chang et al. (2015[6]) reported that IL-6 level in high glucose-induced senescent BM-MSCs was 4 times higher than its level in low glucose-induced senescent BM-MSCs. Since IL-6 plays a key role in SASP (Coppé et al., 2010[9]), therefore higher mRNA level of *IL-6 *in D-ASCs co-culture may be due to their presence in hyperglycemic environment and subsequent induction of senescence. Overall, this data indicates the low potential of D-ASCs to modulate immune responses in inflammatory environment.

TGF-β1 is a multifunctional cytokine, with the ability to inhibit proliferation, differentiation, and activation of immune cells. This cytokine acts as a potential suppressor in regulation of immune homeostasis (Li et al., 2006[30]). In MSCs/T cells co-culture, blocking of TGF-β1 and PGE2 showed that both factors play non-redundant roles in Tregs induction (English et al., 2009[14]). Puissant et al. reported that in supernatant of ASCs/MLR co-culture, IL-10 and TGF-β1 were undetectable (Puissant et al., 2005[40]). In the present study, expression of *TGF-β1* was low in both co-culture groups and there was no significant difference in this cytokine expression level between these groups. 

*TNF-α* is a pro-inflammatory cytokine that contributes to the survival, cell death, differentiation, proliferation and migration (Parameswaran and Patial, 2010[37]). It was observed that in co-culture of BM-MSCs and Wharton jelly MSCs with PBMCs, the level of TNF-α decreased in both groups along with reduction of IFN-γ level (Prasanna et al., 2010[39]). In co-culture of MSCs with PHA/IL-2-activated TCD4^+^ cells, the levels of IFN-γ and TNF-α declined (Tobin et al., 2013[45]). In our previous studies, we observed that presence of MSCs prevented the increment of *TNF-α* expression in MSCs/MLR co-culture (Abolhasani et al., 2018[1]; Hesami et al., 2017[21]). This phenomenon was also observed in the ND-ASCs co-culture group of the present study. However, the expression of this gene remarkably increased on day 4 of D-ASCs co-culture. Studies have revealed that, PGE2 inhibitors increased TNF-α from DC cells and subsequently T cells (Hart et al., 1989[18]). Regarding the role of COX-2 in the PGE2 production, our study results revealed the simultaneous reduction of *COX-2 *expression with increment of *TNF-α* expression in D-ASCs co-culture. It seems that the inability of D-ASCs to inhibit TNF-α expression is a sign of impaired immunomodulatory properties of these cells.

Comparison of D-ASCs and ND-ASCs immunomodulatory properties in co-culture with PBMCs showed reduced expression of COX-2 and increased expression of TNF-α and IL-6 in D-ASCs co-culture compared to ND-ASCs co-culture. Furthermore, in consistence with these findings, the proliferation suppression ability of PBMCs was significantly lower in D-ASCs/MLR co-culture than ND-ASCs/MLR co-culture. These results revealed the impairments of diabetic ASCs in controlling inflammatory conditions, and since diabetes has an inflammatory nature, it can be argued that the acquired or existent impairment in the ASCs of diabetic subjects is responsible for the persistence of inflammatory condition and subsequently stable high blood glucose level of these subjects.

## Funding

This work was supported by Research Council of Kurdistan University of Medical Sciences [1397.140].

## Acknowledgements

The paper is result of a master degree of immunology thesis. Also we would like to thank Dr. Mahbubeh Sabokdast for helping us in providing human adipose tissue samples. 

## Conflict of interest

The authors declare that they have no conflict of interest.

## Figures and Tables

**Table 1:**
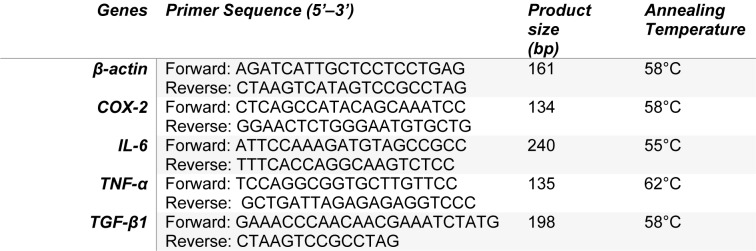
Designed primers sequence, length of amplicons and annealing temperature of the *β-actin, COX-2, IL-6, TNF-α *and* TGF-β1 *genes for evaluation of their mRNA levels with real time PCR
